# Plyometric Jump Training Effects on Maximal Strength in Soccer Players: A Systematic Review with Meta-analysis of Randomized-Controlled Studies

**DOI:** 10.1186/s40798-024-00720-w

**Published:** 2024-05-10

**Authors:** Javier Sanchez-Sanchez, Alejandro Rodriguez-Fernandez, Urs Granacher, José Afonso, Rodrigo Ramirez-Campillo

**Affiliations:** 1 Research Group Planning and Assessment of Training and Athletic Performance, Universidad Pontificia de Salamanca, 37007 Salamanca, Spain; 2 Faculty of Physical Activity and Sports Sciences, VALFIS Research Group, Institute of Biomedicine (IBIOMED), Universidad de León, 24071 León, Spain; 3 Department of Sport and Sport Science, Exercise and Human Movement Science, University of Freiburg, 79102 Freiburg, Germany; 4 Centre of Research, Education, Innovation, and Intervention in Sport (CIFI2D), Faculty of Sport, University of Porto, 4200-450 Porto, Portugal; 5 Exercise and Rehabilitation Sciences Institute, School of Physical Therapy, Faculty of Rehabilitation Sciences, Universidad Andres Bello, 7591538 Santiago, Chile

**Keywords:** Plyometric exercise, Muscle strength, Team sports, Athletic performance, Physical fitness

## Abstract

**Background:**

Maximal strength may contribute to soccer players’ performance. Several resistance training modalities offer the potential to improve maximal strength. During recent years, a large number of plyometric jump training (PJT) studies showed evidence for maximal strength improvements in soccer players. However, a comprehensive summary of the available data is lacking.

**Objective:**

To examine the effects of PJT compared with active, passive or intervention controls on the maximal strength of soccer players, irrespective of age, sex or competitive level.

**Methods:**

To perform a systematic review with meta-analysis following PRISMA 2020. Three electronic databases (PubMed, Web of Science, and SCOPUS) were systematically searched. Studies published from inception until March 2023 were included. A PICOS approach was used to rate studies for eligibility. The PEDro scale was used to assess risk of bias. Meta-analyses were performed using the DerSimonian and Laird random-effects model if ≥ 3 studies were available. Moderator and sensitivity analyses were performed, and meta-regression was conducted when ≥ 10 studies were available for a given comparison. We rated the certainty of evidence using GRADE.

**Results:**

The search identified 13,029 documents, and from these 30 studies were eligible for the systematic review, and 27 for the meta-analyses. Overall, 1,274 soccer players aged 10.7–25.0 years participated in the included studies. Only one study recruited females. The PJT interventions lasted between 5 and 40 weeks (median = 8 weeks), with 1–3 weekly sessions. Compared to controls, PJT improved maximal dynamic strength (18 studies, 632 participants [7 females], aged 12.7–24.5 y; effect size [ES] = 0.43, 95% confidence interval [CI] = 0.08–0.78, *p* = 0.017, impact of statistical heterogeneity [I^2^] = 77.9%), isometric strength (7 studies; 245 participants, males, aged 11.1–22.5 y; ES = 0.58, 95% CI = 0.28–0.87, *p* < 0.001, I^2^ = 17.7%), and isokinetic peak torque (5 studies; 183 participants, males, aged 12.6–25.0 y; ES = 0.51, 95% CI = 0.22–0.80, *p* = 0.001, I^2^ = 0.0%). The PJT-induced maximal dynamic strength changes were independent of participants’ age (median = 18.0 y), weeks of intervention (median = 8 weeks), and total number of training sessions (median = 16 sessions). The certainty of evidence was considered low to very low for the main analyses.

**Conclusions:**

Interventions involving PJT are more effective to improve maximal strength in soccer players compared to control conditions involving traditional sport-specific training.

*Trial Registration* The trial registration protocol was published on the Open Science Framework (OSF) platform in December 2022, with the following links to the project (https://osf.io/rpxjk) and to the registration (https://osf.io/3ruyj).

**Supplementary Information:**

The online version contains supplementary material available at 10.1186/s40798-024-00720-w.

## Key Points

- Plyometric jump training is an easy-to-administer training regime that can be performed on the soccer pitch and may have the potential to improve maximal strength in soccer players.
- Findings from this meta-analysis including 1274 soccer players indicate maximal strength improvements after plyometric jump training, with similar effects for youth and adult players, and with < 8 vs. ≥ 8 weeks or < 16 vs. ≥ 16 sessions

**Supplementary Information:**

The online version contains supplementary material available at 10.1186/s40798-024-00720-w.

## Background

Measures of muscular fitness (i.e., muscle strength, power, local muscular endurance) have been shown to be positively related to markers of health [1–4], and athletic performance [5, 6]. For the context of this systematic review with meta-analysis, maximal strength has been defined as the ability to exert maximal force against an external resistance during a maximal voluntary contraction [7]. Maximal strength is considered a reliable measure of muscular fitness [8] and is commonly assessed through the one-repetition maximum (1-RM) method during a dynamic strength test [6], although alternative assessment techniques (e.g., isometric; isokinetic) are applied as well [9]. Maximal strength depends on factors such as intra- (motor unit recruitment and firing rate) and inter- (agonist-antagonist coordination) muscular coordination as well as muscle cross-sectional area [10]. The improvement in neural and morphological factors related to maximal strength allows athletes to have greater potential for power development [11] and sports-related skills [12]. For example, the development of maximal muscle strength is considered crucial for soccer players to respond to the multiple short-duration and maximal or near-maximal-intensity demands of a match (e.g., sprinting, acceleration, deceleration, jumping, and changes-of-direction) [13–15]. Further, there is evidence showing that maximal strength tests (e.g., load lifted in half squat) are related with linear sprint speed, the main action that precedes a goal in elite [16], semi-professional [14], and youth [17] soccer players. Furthermore, maximal strength (i.e., isokinetic peak torque) can be used to differentiate between soccer players of different practice levels [18].

Although the methods and loads to achieve the best results in maximal strength development have not yet been clarified [19], free weight exercises have been commonly used [7] and a meta-analysis has reported that competitive athletes experience maximal gains when training at an intensity of ~ 85% of the 1-RM [20]. However, factors such as logistical constraints (e.g., reduced access to free weight facilities), the congested schedule of professional soccer players, among others (e.g., injury or reduced performance in young soccer players), may discourage the use of high loads during training for maximal strength development [21]. In this context, plyometric jump training (PJT) presents promising advantages [12, 22] because it can be easily administered on the soccer pitch, is widely applied [23] and does not need much exercise equipment. PJT is characterized through a wide variety of different jump types (e.g., vertical, horizontal, unilateral, bilateral), leveraging body mass as resistance, and necessitating no external load [24]. Additionally, PJT involves rapid muscle eccentric actions during the braking phase immediately followed by rapid concentric actions, mimicking the specific neuromuscular needs commonly encountered in a soccer match, allowing greater transmutation [25–27]. Indeed, previous studies have demonstrated that integrating PJT alongside regular soccer sessions holds promise for enhancing various facets of physical fitness in players of different age, sex and competitive level [28–32]. There is even evidence that PJT can mitigate injury rates [33, 34].

PJT involves repeated jump actions (i.e., multi-jumps) and ground contact times during jumping are either short (i.e., < 250 ms) or long (i.e., > 250 ms), influencing the speed of the muscle–tendon stretch–shortening cycle (SSC) [35]. Muscle actions during the SSC allow an accumulation of elastic energy that facilitates greater muscle power production [36]. Therefore, the SSC stimulates the ability of the neuromuscular system to produce maximal strength and power in the shortest amount of time (i.e., increased rate of force development), with a high transfer to athletic performance [25–27], possibly due to improved neural activation and enhanced motor coordination [23, 37]. PJT may also increase skeletal muscle fibre contraction force and cross-sectional area [38, 39], pennation angle [40], among other neuromuscular adaptations [23], all linked to training-induced maximal strength improvements [41]. Indeed, PJT has the potential to improve maximal strength. For example, a meta-analysis [12] including 15 PJT studies showed improved maximal strength with an effect size (ES) = 0.97 vs 0.11 in trained compared to controls, respectively. The results of these analyses indicate that the PJT effects on maximal strength do not depend on the sport discipline (ES = 0.87, volleyball; ES = 0.41, basketball; ES = 0.80, body building; ES = 0.80, rowing; ES = 0.50, swimming; ES = 0.80, American football). However, no soccer-related studies were included.

Moreover, most studies investigating PJT effects in soccer involved only small sample sizes (i.e., mode n = 10) [24, 35, 42] which is a common problem in the sport science literature using highly trained athletes [43]. In an attempt to address the challenge of small sample sizes in elite sports, systematic reviews with and without meta-analyses have been conducted in adult male [44] and female players [32], as well as in young players [38, 39]. However, not all studies [28, 44] agree with the beneficial effects of PJT on measures of maximal strength in soccer. A previous systematic review showed no PJT effects on maximal strength in adult male soccer players [44], while another systematic review showed a significant effect in youth soccer players [28]. Of note, the transfer of findings from adult male to youth players (including females) appears inappropriate due to maturational processes taking place in youth such as rapid increases in stature, potential temporary disruption in motor co-ordination also known as adolescent awkwardness, large increases in fat-free mass due to hormonal changes, and changes in muscle–tendon architecture [45–47]. These factors may influence the muscle strength responsiveness to PJT [45, 48–56]. Currently, there is no study available that has contrasted youth vs. adult soccer players’ responsiveness to PJT.

To account for the previous limitations (e.g., low sample size), a meta-analysis may help practitioners to extract evidence-based information from the available literature for PJT implementation in soccer [57]. Additionally, a systematic review with meta-analysis may detect gaps and limitations in the PJT literature (e.g., lack of studies addressing the chronobiological response of maximal strength to PJT), providing valuable information for scientists and practitioners about future research avenues. Such an approach seems timely, considering that the yearly rate of PJT-related publications has increased 25-fold between 2000 and 2017 [24], with soccer-related studies at a rate of around 100 per year [58]. Therefore, the primary aim of this systematic review with meta-analysis was to examine the effects of PJT compared with active, passive or intervention controls on the maximal strength in soccer players of any age, sex or competitive level.

## Methods

### Procedures

A systematic review with meta-analysis was conducted following the guidelines of the updated Preferred Reporting Items for Systematic Reviews and Meta-Analyses (PRISMA) 2020 [59]. The protocol for this systematic review with meta-analysis was published on the Open Science Framework (OSF) platform in December 2022, with the following links to the project (https://osf.io/rpxjk) and to the registration (https://osf.io/3ruyj).

### Literature Search: Administration and Update

We considered recommendations from the two most comprehensive scoping reviews that previously examined the PJT literature [24, 31]. Briefly, a systematic literature search of three electronic databases (PubMed, Web of Science, and SCOPUS) was conducted. Studies published from inception and until March 2023 were included. The search strategy (code line) for each database is described in the Additional file 1: Table S1. In selecting studies for inclusion, a review of all relevant titles was conducted before the examination of the abstracts and full-texts. Two authors (RRC and JSS) independently screened the titles, abstracts, and/or full-texts of the retrieved studies. During the search and review process, potential discrepancies between the two authors regarding inclusion and exclusion criteria (e.g., intervention adequacy) were resolved through discussion with a third author (ARF)

### Inclusion and Exclusion Criteria

A PICOS (participants, intervention, comparators, outcomes, and study design) approach was used to rate studies for eligibility [60]. Table 1 indicates the inclusion/exclusion criteria. Of note, the decision to determine the minimal effective PJT duration (weeks) for the improvement of maximal strength (i.e., ≥ 3 weeks) was based on the results of previous systematic reviews [12, 24, 35]. Additionally, only original studies in peer-reviewed and full-text format were eligible to be included in this meta-analysis.

**Table 1:** Selection criteria

Category	Inclusion criteria	Exclusion criteria
Population	Soccer players, with no restrictions on their fitness, competitive level, age, or sex	Participants with health problems (e.g., injuries, recent surgery), precluding participation in a plyometric-jump training program. No exclusion was applied based on disability (e.g., para-soccer) as long as the participants were able to complete a plyometric-jump training programme without restrictions on intensity
Intervention	A plyometric-jump training program, with a minimal duration of ≥ 3 weeks*, which included unilateral and/or bilateral jumps, which commonly utilize a pre-stretch or countermovement stressing the stretch–shortening cycle	Exercise interventions not involving plyometric-jump training (e.g., upper-body plyometrics only training interventions) or exercise interventions involving plyometric jump training programs representing less than 50% of the total training load (i.e., volume, e.g., number of exercises) when delivered in conjunction with other training interventions (e.g., high-load resistance training)
Comparator	Active control group (i.e., soccer players participating in regular training schedules) Studies comparing different plyometric-jump training approaches (e.g., different intensity) without active control group, or passive control group (i.e., soccer players non-participating in any regular training during intervention) were also considered, as well as intervention control groups (e.g., soccer players involving alternative training methods such as high-load resistance training)	Absence of control group
Outcome	At least one measure related to maximal strength (e.g., 1RM squat) before and after the training intervention	Lack of baseline and/or follow-up data related to strength
Study design	Multi-arm trials	Single-arm trials/observational studies

*Justification in the main text

We excluded books, book chapters, and congress abstracts, as well as cross-sectional studies and review papers. The following studies were excluded: retrospective studies, prospective studies (e.g., relation between bone density at the end of PJT, and at several years of follow-up), studies reported in proceedings (only abstract available), special communications, letters to the editor, invited commentaries, errata, and studies of doubtful quality or unclear peer-review process [61]. In the case of detraining studies, these were considered for inclusion if they involved a training period prior to a detraining period. When a comparator group was included in the studies, we did not consider a minimum number of participants per group as an inclusion/exclusion criterion, although case reports were excluded.

### Data Item Extraction and Management

The effects of PJT compared to active (e.g., young soccer players, female soccer players), passive (e.g., soccer players non-participating in any regular training during intervention) and/or intervention (e.g., soccer players involving alternative training methods such as high-load resistance training) controls on maximal strength were assessed. Measures of maximal strength included (but were not limited to) different specific tests (e.g., squat; leg press). The 1-RM has previously shown moderate to strong levels of reliability (intra-class correlation coefficient = 0.64–0.99; coefficient of variation = 0.5–12.1%) across a range of populations [8], which is essential to ensure strong consistency between the analysed studies within a meta-analysis [60].

Pre- and post-intervention, means and standard deviations of the dependent variables were extracted from the included studies using Microsoft Excel® (Microsoft Corporation, Redmond, WA, USA). For studies reporting values other than means and standard deviation (e.g., median, range, interquartile range, standard error values), conversion was applied as previously recommended [62–64]. Appropriate statistical software was used for different data formats (Comprehensive Meta-Analysis Software, Version 2, Biostat, Englewood, NJ, USA). When the required data were not clearly or completely reported, the authors of the respective studies were contacted for clarification purposes. If no response was obtained from the authors (after two attempts, with a between-attempts waiting time of 72 h) or the authors did not provide the requested data, the study outcome was excluded from further analysis. According to our registered protocol, four studies were excluded because they did not respond to our author queries and data were not sufficiently reported for the purpose of a meta-analysis, three full texts were excluded from the meta-analysis because maximal strength data were reported in combined form for both control and experimental groups (as the authors noted no difference between experimental and control groups) [65, 66] or 1-RM was measured only in the experimental group [67]. When data were displayed in a figure and no numerical data were provided by the authors, validated (*r* = 0.99, *p* < 0.001) [60] software (WebPlotDigitizer, version 4.5; https://apps.automeris.io/wpd/) was used to derive numerical data from the respective figures. One author (JSS) performed data extraction and a second author (ARF) provided confirmation, and any discrepancies between them (e.g., mean value for a given outcome) were resolved through discussion with a third author (RRC).

### Risk of Bias of the Included Studies

The Physiotherapy Evidence Database (PEDro) scale was used to assess the risk of bias and methodological quality of the included studies. The validity and reliability of the PEDro scale have been established previously [68–70]. Moreover, the PEDro scale is the most frequently used metric in the PJT literature [35, 71, 72]. Despite being termed a “methodological quality” scale, its items mostly assess factors related to the risk of bias of studies. Accordingly, it helps to make comparisons between meta-analyses. Considering that it is not possible to satisfy all scale items in PJT interventions [73] and as outlined in previous systematic reviews in the sub-field of PJT, the overall risk of bias of PJT studies was interpreted using the following convention [31, 32, 72, 73]: ≤ 3 points was considered as “poor” quality (i.e., high risk of bias), 4–5 points was considered as “moderate” quality, while 6–7 points and 8–10 points was considered as “good” and “excellent” quality, respectively. For practical purposes and given the nature of the research field, we considered studies with ≥ 6 points to have low risk of bias. Two reviewers (JSS and ARF) independently rated/confirmed each study. Any ratings that yielded different results between two reviewers were further adjudicated by a third reviewer (RRC).

### Summary Measures, Synthesis of Results, and Risk of Publication Bias

Meta-analyses can be computed with as little as two studies [74]. However, we performed our analyses if ≥ 3 studies were available considering the reduced number of participants in PJT interventions [1, 75, 76]. Means and standard deviations from pre- and post-values were taken to compute ES (i.e., Hedges' *g*) for maximal strength in the PJT and active, passive or intervention control groups. Data were standardised using post-intervention standard deviation values. The DerSimonian and Laird random-effects model was used to account for differences between studies that might affect the PJT effects [77, 78]. The ES values are presented with 95% confidence intervals (95% CIs). Calculated ES were interpreted using the following scale: < 0.2 trivial, 0.2–0.6 small, > 0.6–1.2 moderate, > 1.2–2.0 large, > 2.0 very large [79]. However, in strength and conditioning research studies with ES values ≥ 3.0 (improvement of ≥ 3.0 standard deviations from the mean) are unlikely after most interventions, and were considered outliers [80]. In studies including more than one intervention group, the sample size in the control group was proportionately divided to facilitate comparisons across multiple groups [81]. The impact of study heterogeneity was assessed using the *I*^2^ statistics, with values of < 25%, 25–75%, and > 75% representing low, moderate, and high levels of heterogeneity, respectively [82]. The risk of publication bias was explored for continuous variables (≥ 10 studies per outcome) [83–85] using the extended Egger’s test [84]. All analyses were carried out using the Comprehensive Meta-Analysis Software (Version 2, Biostat, Englewood, NJ, USA). Statistical significance was set at *p* ≤ 0.05.

### Additional Analyses

#### Subgroup Analyses

As adaptive responses to PJT programs may be affected by the individual’s age and sex [56, 86, 87], these potential sources of heterogeneity are likely to influence the effects of training and were therefore selected a priori.

#### Single Training Factor Analyses

Single training factor analyses were computed for the programming parameters duration (intervention duration and total number of training sessions) [88] and training frequency (number of weekly exercise sessions) [12], based on the reported impact of these variables on adaptations following PJT.

When appropriate, subgroup analyses and single training factor analyses were analysed using the median split technique [87, 89, 90]. The median was calculated if at least three studies provided data for a potential moderator. Of note, when two experimental groups (with the same information for a given moderator) were included in a study, only one of the groups was considered to avoid an augmented influence of the study on the median calculation. In addition, instead of using a global median value for a given moderator (e.g., median age, derived from all included studies), median values were calculated considering only those studies that provided data for the analysed outcome. When the median split technique was found not to be appropriate, a logically defensible rationale was used for subgroup analysis.

#### Sensitivity Analyses

We performed sensitivity analyses to assess the robustness of the summary estimates (e.g., *p*-value, ES, *I*^2^). To examine the effects of each result from each study on the overall findings, results were analysed with each study deleted from the model (automated leave-one-out analysis).

#### Meta-regression

A multivariate DerSimonian and Laird random-effects model meta-regression was conducted to verify if any of the training variables (frequency, duration, and total number of sessions) explained the effects of PJT on the maximal strength. The computation of meta-regression was performed with at least 10 studies per covariate [91].

#### Certainty of Evidence

Two authors (JA and RRC) rated the certainty of evidence (i.e., high; moderate; low; very low) using the Grading of Recommendations, Assessment, Development and Evaluation (GRADE) [92–94]. The evidence started at a high level of certainty (per outcome), but was downgraded based on the following criteria: (i) *Risk of bias in studies*: judgments were downgraded by one level if the median PEDro scores were moderate (< 6) or by two levels if they were poor (< 4); (ii) *Indirectness*: low risk of indirectness was attributed by default due to the specificity of populations, interventions, comparators and outcomes being guaranteed by the eligibility criteria; (iii) *Risk of publication bias*: downgraded by one level if there was suspected publication bias; (iv) *Inconsistency*: judgments were downgraded by one level when I^2^ was high (> 75%); (v) *Imprecision*: one level of downgrading occurred whenever < 800 participants were available for a comparison [95] and/or if there was no clear direction of the effects. When both were observed, certainty was downgraded by two levels.

#### Adverse Effects

Considering the potential adverse health effects derived from the inadequate implementation of PJT interventions, a qualitative analysis of such potential effects was included.

## Results

### Study Selection

**Fig. 1:**
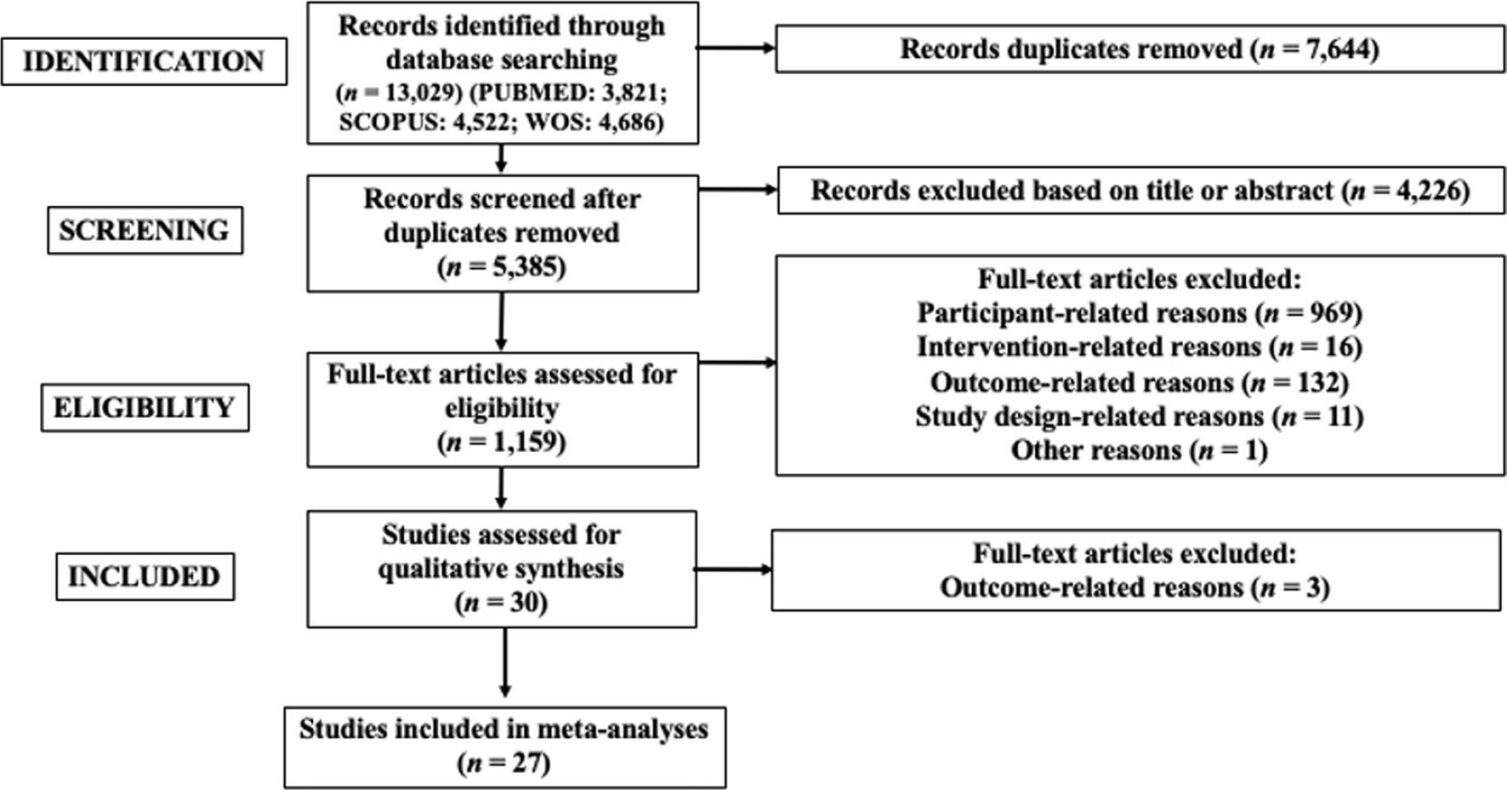
Study inclusion and exclusion selection process

The search process in the databases identified 13,029 studies. Figure 1 provides a flow chart illustrating the study selection process. Duplicate studies were removed (*n* = 7644). After study titles and abstracts were screened, 4226 studies were removed and 1159 full texts were screened. Finally, 30 studies (all written in English) were considered eligible to be included in this systematic review [65–67, 96–122]. Three full texts were excluded from the meta-analysis because maximal strength data were reported in combined form for both control and experimental groups (as the authors noted no difference between experimental and control groups) [65, 66] or 1-RM was measured only in the experimental group [67].

### Risk of Bias of the Included Studies

According to the PEDro checklist results (Table 2), the median (i.e., non-parametric) score was 5 (some risk of bias-moderate quality), with 17 studies attaining 4–5 points (some risk of bias-moderate quality) and 13 studies attaining 6–7 points (low risk of bias-high quality). For studies that analyzed the effects of PJT on dynamic maximal strength, the median score was 6 (low risk of bias-high quality), with six studies attaining 4–5 points (some risk of bias-moderate quality) and seven studies attaining 6–7 points (low risk of bias-high quality). For the studies that analyzed the effect of PJT on maximal voluntary isometric strength, the median score was 5 (some risk of bias-moderate quality), with four studies attaining 4–5 points (some risk of bias-moderate quality) and three studies attaining 6–7 points (low risk of bias-high quality). The studies that analyzed the effects of PJT on peak isokinetic torque obtained a median score of 5 (some risk of bias-moderate quality), with three studies attaining 5 points (some risk of bias-moderate quality) and two studies attaining 6–7 points (low risk of bias-high quality).

**Table 2:** Rating of studies according to the Physiotherapy Evidence Database (PEDRo) scale

	1	2	3	4	5	6	7	8	9	10	11	Score^a^	Study quality
Al Ameer [90]	1	0	0	1	0	0	0	1	1	0	1	4	Moderate
Brito et al. [91]	1	1	0	0	0	0	0	1	1	1	1	5	Moderate
Coratella et al. [92]	1	1	1	0	0	0	1	1	1	1	1	7	High
Faude et al. [93]	1	1	0	1	0	0	0	0	1	1	1	5	Moderate
Franco-Márquez et al. [94]	1	0	0	1	0	0	0	1	1	1	1	5	Moderate
Gauffin et al. [99]	1	1	0	0	0	0	0	1	1	0	1	4	Moderate
Gauffin et al. [98]	1	1	0	0	0	0	0	1	1	1	1	5	Moderate
Hammami et al. [96]	1	1	0	0	0	0	0	1	1	1	1	5	Moderate
Hammami et al. [95]	1	1	0	1	0	0	0	1	1	1	1	6	High
Hasan et al. [115]	1	1	0	1	0	0	0	0	1	1	1	5	Moderate
Ita & Guntoro [114]	1	1	0	1	0	0	0	1	1	1	1	6	High
Keiner et al. [97]	1	1	0	1	0	0	0	0	0	1	1	4	Moderate
Lehnert et al. [119]	1	1	0	0	1	1	1	0	1	1	1	7	High
Lockie et al. [113]	1	1	0	1	0	0	0	1	1	0	1	5	Moderate
Makhlouf et al. [100]	1	1	0	1	0	0	0	1	1	1	1	6	High
McKinlay et al. [111]	1	0	0	1	0	0	0	1	1	1	1	5	Moderate
Mendiguchia et al. [112]	1	1	0	1	0	0	0	0	1	1	1	5	Moderate
Michailidis et al. [110]	1	1	0	1	0	0	0	0	1	1	1	5	Moderate
Moore et al. [118]	1	0	0	1	0	0	0	1	1	1	1	5	Moderate
Negra et al. [109]	1	1	0	1	0	0	0	0	1	1	1	5	Moderate
Raedergard et al. [102]	1	1	0	1	0	0	0	1	1	1	1	6	High
Ramirez-Campillo et al. [116]	1	1	1	1	0	0	0	1	1	1	1	7	High
Rodriguez-Rossel et al. [108]	1	1	0	1	0	0	0	1	1	1	1	6	High
Rodriguez-Rossel et al. [107]	1	1	0	1	0	0	0	1	1	1	1	6	High
Ronnestad et al.[106]	1	1	0	1	0	0	0	1	1	1	1	6	High
Siegler et al. [105]	1	1	0	1	0	0	0	1	1	1	1	5	Moderate
Spineti et al. [117]	1	1	0	1	0	0	0	1	1	1	1	6	High
Vaczi et al. [104]	1	1	0	0	0	0	0	1	1	1	1	5	Moderate
Vera-Asaoka et al. [103]	1	1	0	0	0	0	1	1	1	1	1	6	High
Zghal et al. [101]	1	1	1	0	1	1	0	0	1	1	1	7	High

A detailed explanation for each PEDro scale item can be accessed at https://www.pedro.org.au/english/downloads/pedro-scale. In brief: item 1, eligibility criteria were specified; item 2, participants were randomly allocated to groups; item 3, allocation was concealed; item 4, the groups were similar at baseline; item 5, there was blinding of all participants regarding the plyometric jump training programme being applied; item 6, there was blinding of all coaches responsible for the application of plyometric jump training programme regarding its aim toward the improvement of maximum strength; item 7, there was blinding of all assessors involved in measurement of maximum strength; item 8, measures of maximum strength were obtained from more than 85% of participants initially allocated to groups; item 9, all participants for whom maximum strength value was available received the treatment or control condition as allocated or, data for maximum strength were analysed by “intention to treat”; item 10, the results of between-group statistical comparisons are reported for maximum strength value; and item 11, point measures and measures of variability for maximum strength are provided

^a^From a possible maximal score of 10

### Study Characteristics

The participant characteristics and the PJT programs of the included studies are detailed in Table 3. In the 30 studies included in the systematic review 1,274 soccer players were included (624 in the intervention groups; 650 in control groups), with an age range of 10.7–25.0 years (14 studies recruited participants < 18 years). Intervention control groups (e.g., involving alternative training methods such as high-load resistance training) were considered a priori for moderator analyses [97, 102, 103, 109–112, 114, 118, 123]. However, study heterogeneity (e.g., moderate-load RT; high-load RT; weightlifting; resisted sprinting; free sprinting; functional training) precluded such analyses.

**Table 3:** Descriptive characteristics of participants and plyometric jump training interventions

	Rand	n	Sex	Age (y)	Body mass (kg)	Height (cm)	SPT	Fit	Fr	Weeks (n)	NTJ (n)
Al Ameer [90]	No	30:30	M	18–24	NR	NR	NR	NR	2	12	2,880
Brito et al. [91]	Yes	12/12:21/12	M	20.0/19.9	71.6/72.2	176/180	No	N	2	9	360
Coratella et al. [92]	Yes	16/16:16	M	18–25	73.0	178	NCR	Mo	2	8	800/656
Faude et al. [93]	Yes	8:8	M	22.5	76.8	179	NCR	Mo	2	7	360
Franco-Márquez et al. [94]	No	22:22	M	14.7	60.3	171	No	Mo	2	6	1,818
Gauffin et al. [99]	Yes	36:18	M	20.0	NR	NR	NR	Mo	3	10	900
Gauffin et al. [98]	Yes	20/20:50	M	21.0	NR	NR	No	Mo	2	10	72/66
Hammami et al. [96]	Yes	16:12/16	M	16.0	59.3	178	NR	Mo	2	8	480
Hammami et al. [95]	Yes	14/14:12	M	15.7–16.0	58.3–59.0	177/175	NCR	Mo	2	8	1,440/722
Hasan et al. [115]	Yes	30:30/30	M	20.6	64.7	173	NR	Mo	3	6	1,440
Ita & Guntoro [114]	Yes	15:15	M	21.3	66.1	169	NR	N	NR	6	NR
Keiner et al. [97]	Yes	11:12/11/14	M	17–18	73.0	178	NR	Mo	2	40	2,880–3,840
Lehnert et al. [119]	Yes	6:6	M	17.8	74.5	180	NCR	H	2–3	5	NR
Lockie et al. [113]	Yes	9:/9/9/9	M	23.1	83.1	182	NCR	Mo	2	6	834
Makhlouf et al. [100]	Yes	20/21:16	M	11.1/11.3	36.9/36.2	145/150	No	Mo	2	8	1,826
McKinlay et al. [111]	No	13:14/14	M	12.6	47.2	158	No	Mo	3	8	3,438
Mendiguchia et al. [112]	Yes	27:24	M	22.7	71.6	175	NCR	Mo	1	7	278
Michailidis et al. [110]	Yes	24:21	M	10.7	42.5	147	No	Mo	2	12	> 1,560
Moore et al. [118]	No	8:7	M-F	20.6	68.7	170	No	Mo	3	11	2,748
Negra et al. [109]	Yes	11:11/12	M	12.7	45.9	156	NCR	Mo	2	12	> 1,344
Raedergard et al. [102]	Yes	11:10	M	22.6	82.5	182	Yes	Mo	2	6	1,032
Ramirez-Campillo et al. [116]	Yes	25/24:24	M	13.9/13.1	46.7/47.2	153	No	Mo	2	7	906
Rodriguez-Rossel et al. [108]	Yes	15:15	M	12.7	47.6	158	No	Mo	1	6	90
Rodriguez-Rossel et al. [107]	Yes	10:10/10	M	24.5	74.4	176	No	Mo	1–2	6	90
Ronnestad et al.[106]	Yes	8:7/6	M	23.0	73.5	180	Yes	H	2	7	672
Siegler et al. [105]	No	17:17	F	16.5	61.5	167	No	N	1–2-3	10	1,046
Spineti et al. [117]	Yes	10:12	M	18.4	70.2	180	NCR	H	2	8	1,440
Vaczi et al. [104]	Yes	12:12	M	21.9	75.9	180	Yes	Mo	2	6	925
Vera-Asaoka et al. [103]	Yes	16/22:16/22	M	11.2/14.4	36.8/54.7	143/163	No	Mo	2	7	840
Zghal et al. [101]	Yes	9/14:8	M	14.5	60.2/59.3	172/171	No	Mo	1	7	462/924

*F* Female; *Fit* Fitness; *Fr* Frequency of PJT as number of sessions per week; *H* High; *M* Male; *Mo* Moderate; *n* Values to the left and right from “:” denotes the number of participants in the intervention and control groups (respectively), and the values separated from “/” denotes the number of group participants when two or more groups were included as either intervention or controls. For example, “11:12/11/14” denotes that one intervention group, with 11 participants, was included, and that three control groups were included, involving 12, 11, and 14 participants, respectively. *NCR* Not clearly reported; *No* Normal; *NR* Not reported; *NTJ* number of total jumps; *Rand* Randomised; *SPT* Systematic jump training experience before intervention

The testing procedures for maximal strength among studies are described in Table 4. Broadly, testing procedures were categorized as maximal dynamic, maximal isometric, and peak isokinetic torque tests. Briefly, most (n = 20) studies included dynamic 1-RM to 5-RM squat tests, isometric tests (n = 7), isokinetic tests (n = 6), or included a combination of ≥ 2 types of the mentioned maximal strength test categories.

**Table 4:** Maximal strength testing procedures

	Dynamic maximal strength								Isometric maximal strength							Isokinetic peak torque										
	a	b	c	d	e	f	g	h	i	j	k	l	m	n	o	p	q	r	s	t	u	v	w	x	y	z
Al Ameer [90]	x																									
Brito et al. [91]				x												x	x		x	x	x	x				
Coratella et al. [92]				x												x		x			x		x			
Faude et al. [93]				x						x	x	x	x													
Franco-Márquez et al. [94]				x																						
Gauffin et al. [99]																x			x							
Gauffin et al. [98]																x			x							
Hammami et al. [96]				x																						
Hammami et al. [95]				x																						
Hasan et al. [115]									x																	
Ita & Guntoro [114]										x																
Keiner et al. [97]				x																						
Lehnert et al. [119]																x	x		x	x						
Lockie et al. [113]					x			x																		
Makhlouf et al. [100]									x					x												
McKinlay et al. [111]									x						x	x								x		
Mendiguchia et al. [112]																x	x		x	x					x	x
Michailidis et al. [110]				x																						
Moore et al. [118]						x																				
Negra et al. [109]				x																						
Raedergard et al. [102]				x																						
Ramirez-Campillo et al. [116]							x																			
Rodriguez-Rossel et al. [108]				x																						
Rodriguez-Rossel et al. [107]				x																						
Ronnestad et al.[106]				x																						
Siegler et al. [105]		x	x	x																						
Spineti et al. [117]				x																						
Vaczi et al. [104]									x																	
Vera-Asaoka et al. [103]							x																			
Zghal et al. [101]									x						x											

a, 1RM leg press; b, 1RM leg extension; c, 1RM calves; d, 1RM squat; e, 3RM squat; f, 4RM squat; g, 5RM squat; h, 3RM/body mass squat; i, knee extension; j, leg press bilateral; k, Leg press right leg; l, Leg press left leg; m, leg press, slope of the force–time relationship; n, Back extensor; o, Highest rate at which torque developed; p, Peak Torque, Quadriceps (knee extensor)-Concentric Dominant (right) leg; q, Peak Torque, Quadriceps (knee extensor) -Concentric non-Dominant (left) leg; r, Quadriceps-eccentric Dominant leg; s, Hamstring (knee flexors)-Concentric Dominant leg; t, Hamstring (knee flexors)-Concentric non-Dominant leg; u, Hamstring-Eccentric Dominant leg; v, Hamstring-Eccentric non-Dominant leg; w, Eccentric hamstrings:concentric quadriceps peak torque ratio; x, Peak torque development, quadriceps (knee extensors) dominant (right) leg; y, Concentric hamstring:quadriceps peak torque ratio dominant; z, Concentric hamstring:quadriceps peak torque ratio dominant

### Results from the Meta-analysis

#### Maximal Dynamic Strength

**Fig. 2:**
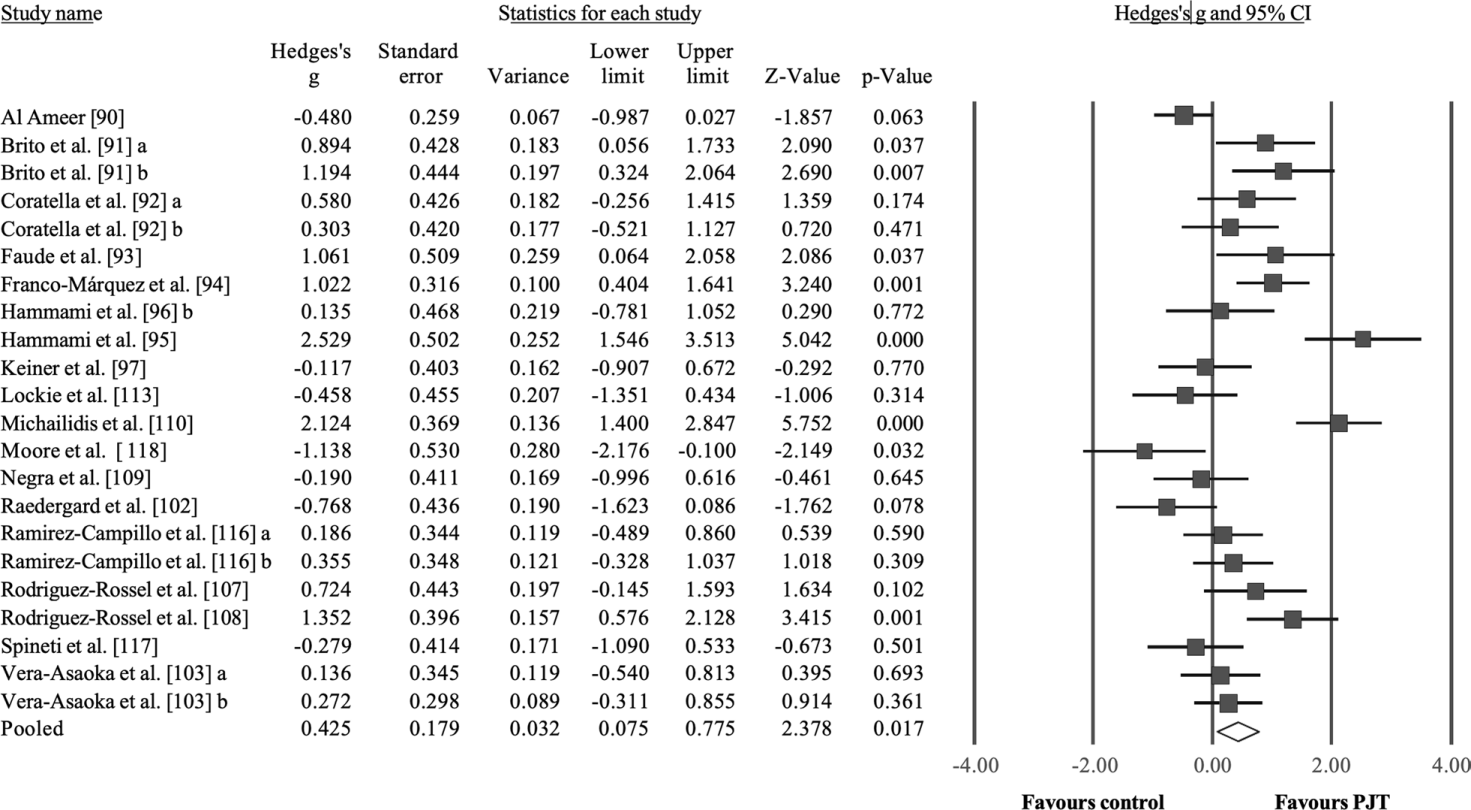
Forest plot illustrating plyometric jump training (PJT)-related improvements of maximal dynamic strength (e.g., 1-RM) in comparison to active and passive controls groups. Forest plot values are shown as effect sizes (Hedges’ g) with 95% confidence intervals (CI). Black squares: individual studies. The size represents the relative weight. White rhomboid: summary value. ^a^ and ^b^ denote different experimental groups used in the same study

Eighteen studies were used to meta-analyze the PJT effects on dynamic maximal strength, examining a total of 632 participants, 342 included in the intervention groups (22 groups) and 290 in the control groups (18 groups). Regarding the 18 control groups, 13 groups were involved in regular soccer training (active control group), five groups were involved in strength training (intervention control group) and one group was classified as passive control since the study was carried out during the off-season [98]. Seventeen studies involved male soccer players and one study used male and female soccer players [118], aged < 18 years in 9 studies (9 studies recruited participants 18–25 years). Training duration in the intervention and control groups ranged from 6 to 40 weeks, with a median value of 8 weeks. The frequency of weekly training sessions ranged from 1 to 3, although most studies (15 studies) applied 2 training sessions per week. Results (Fig. 2) showed a significant effect for the PJT groups compared to the control groups: ES = 0.61, 95% CI = 0.22 to 1.00, *p* = 0.002. However, after two outlier study-groups were removed [101, 109], the results changed to ES = 0.43, 95% CI = 0.08 to 0.78, *p* = 0.017, *I*^2^ = 77.9%, Egger test two-tailed = 0.457. In both cases, despite an effect favouring the PJT groups, the large 95% CIs suggested heterogeneous results. After the sensitivity analyses (automated leave-one-out analysis), the robustness of the summary estimates (e.g., *p*-value, ES) was confirmed.

#### Maximal Voluntary Isometric Strength

**Fig. 3:**
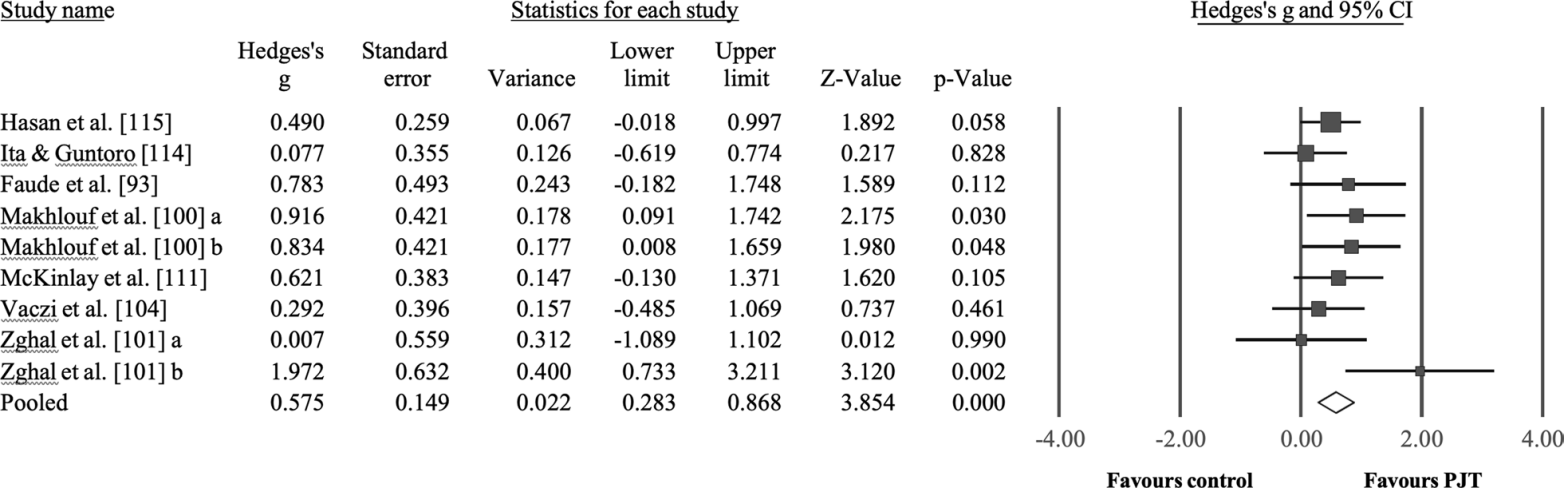
Forest plot illustrating plyometric jump training (PJT)-related improvements of maximal voluntary isometric strength (e.g., N) in comparison to active and passive controls. Forest plot values are shown as effect sizes (Hedges’ g) with 95% confidence intervals (CI). Black squares: individual studies. The size represents the relative weight. White rhomboid: summary value. ^a^ and ^b^ denote different experimental groups used in the same study

Seven studies were considered to analyze the effect of PJT on isometric maximal strength, including a total of 245 participants, with 142 included in the intervention groups (9 groups) and 103 in the control groups (7 groups). Six control groups performed regular soccer training (active control group) and one passive group performed no training (unclear if this was done during the off-season) [118]. All participants were male soccer players aged 11.1–22.5 years (3 studies recruited participants < 18 years). Training duration in the intervention and control groups ranged from 6 to 8 weeks, with a frequency of weekly training sessions ranging from 1 to 3. Results (Fig. 3) showed a significant effect for the PJT groups compared to the control groups: ES = 0.58, 95% CI = 0.28–0.87, *p* < 0.001, *I*^2^ = 17.7%. After the sensitivity analyses (automated leave-one-out analysis), the robustness of the summary estimates (e.g., *p*-value, ES) was confirmed.

#### Peak Isokinetic Torque

**Fig. 4:**
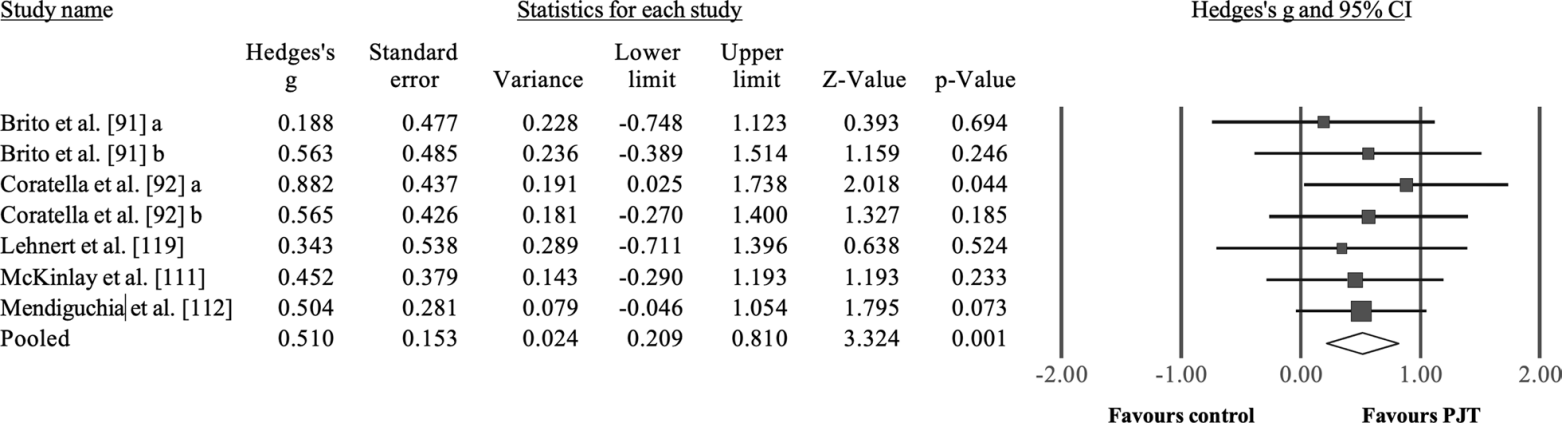
Forest plot illustrating plyometric jump training (PJT)-related improvements of peak isokinetic torque (e.g., N.m-1) in comparison to active, passive and intervention controls. Forest plot values are shown as effect sizes (Hedges’ g) with 95% confidence intervals (CI). Black squares: individual studies. The size represents the relative weight. White rhomboid: summary value. ^a^ and ^b^ denote different experimental groups used in the same study

The effects of PJT on peak isokinetic torque were analyzed in 5 studies, involving 183 participants, with 102 participants from the intervention groups (7 groups) and 81 from the control groups (5 groups). Of the control groups, 3 groups performed regular soccer training (active control group), one group did not perform any training because the study was conducted during the off-season period (passive control group) [92], and one group performed high-resistance training (intervention control group) [119]. All studies involved male soccer players, aged 12.6–25.0 years (2 studies recruited participants < 18 years). Training duration in the intervention and control groups ranged from 5 to 9 weeks, with a median value of 8 weeks. The frequency of weekly training sessions ranged from 1 to 3. Results (Fig. 4) showed a significant effect for the PJT groups compared to the control groups: ES = 0.51, 95% CI = 0.22 to 0.80, *p* = 0.001, *I*^2^ = 0.0%. After the sensitivity analyses (automated leave-one-out analysis), the robustness of the summary estimates (e.g., p-value, ES) was confirmed.

#### Moderator Analyses

Regarding participants’ age (median age = 18.0 y), PJT-induced maximal dynamic strength changes were similar for adults (8 groups; ES = 0.49, 95% CI = 0.07–0.91; *p* = 0.021; *I*^2^ = 46.0%) compared to younger participants (9 groups; ES = 0.37, 95% CI = 0.04–0.69; *p* = 0.026; *I*^2^ = 46.1%), with a between-moderator category *p* = 0.650.

Regarding PJT weeks of intervention (median duration = 8 weeks), PJT-induced maximal dynamic strength changes were similar for longer (5 groups ≥ 8 weeks; ES = 0.57, 95% CI = −0.01 to 1.14; *p* = 0.053; *I*^2^ = 63.4%) compared to shorter interventions (12 groups < 8 weeks; ES = 0.35, 95% CI = 0.08–0.62; *p* = 0.011; *I*^2^ = 30.0%), with a between-moderator category *p* = 0.503.

Regarding total number of PJT sessions (median = 16 sessions), PJT-induced maximal dynamic strength changes were similar after interventions involving higher (6 groups ≥ 16 sessions; ES = 0.43, 95% CI = −0.12 to 0.98; *p* = 0.125; *I*^2^ = 65.9%) compared to lower number of sessions (11 groups < 16 sessions; ES = 0.40, 95% CI = 0.13–0.67; *p* = 0.003; *I*^2^ = 24.7%), with a between-moderator category *p* = 0.932.

Other moderator analyses were precluded due to a reduced number of studies (n < 3) available for each of the moderator categories.

#### Meta-regression

The meta-regression analysis (17 groups) was computed for maximal dynamic strength (e.g., 1-RM) including as potential effect modifiers the i) participants' chronological age (years), ii) PJT total duration (weeks), and iii) PJT total sessions (n). None of the variables in the model explained the effects of PJT on maximal dynamic strength (*p* = 0.299–0.744).

### Certainty of Evidence

According to the GRADE assessment (Table 5), the certainty of evidence was considered low to very low for the main analyses.

**Table 5:** GRADE analyses

Outcomes (PJT vs active, passive or intervention controls)	Studies and PSS	Risk of bias in studies	Risk of publication bias	Inconsistency	Imprecision	Certainty of evidence
Maximal dynamic strength	18, n = 661	No downgrading (median PEDro score: 6)	No downgrading. No evidence of publication bias after removal of two outliers*	Downgraded by one level (*I*^2^ = 82.2% and 77.9%, with and without outliers, respectively)	Downgrade by two levels: (i) < 800 participants; (ii) small mean effect favouring PJT, but distribution of 95% CIs suggests no clear direction of effects	⊕ , Very low
Maximal voluntary isometric strength	7, n = 245	Downgrade by one level (median PEDro score: 5)	–	No downgrading (*I*^2^ = 17.7%)	Downgrade by one level: (i) < 800 participants; (ii) small effect favouring PJT	⊕ ⊕ , Low
Maximal isokinetic strength	5, n = 183	Downgrade by one level (median PEDro score: 5)	–	No downgrading (*I*^2^ = 0.0%)	Downgrade by one level: (i) < 800 participants; (ii) small effect favouring PJT	⊕ ⊕ , Low

i) *Risk of bias in studies*: downgraded by one level if the median PEDro scores were < 6 or by two levels if < 4; ii) *Indirectness*: considered low due to eligibility criteria; iii) *Risk of publication bias*: assessed when ≥ 10 studies were available; downgraded by one level if suspected risk of publication bias; iv) *Inconsistency*: downgraded by one level when the impact of statistical heterogeneity (*I*^2^) was high (> 75%); v) *Imprecision*: downgraded by one level when < 800 participants were available for a comparison or if there was no clear direction of the effects; accumulation of both resulted in downgrading by two levels

*GRADE* Grading of recommendations assessment, development and evaluation; *PJT* Plyometric jump training; *PSS* pooled sample size

*An explanation of outliers’ identification and treatment procedures is provided in the text (Sects. "Summary measures, synthesis of results, and risk of publication bias". and “Maximal dynamic strength”.)

### Adverse Effects

Six studies indicated that 36 players (2.8% of total participants) did not complete the intervention studies due to adverse health events (e.g., injury; illness) [65, 66, 99, 100, 111, 115] without better clarification if events occurred during interventions. The remaining studies did not report soreness, pain, fatigue, injury, damage, or related adverse health events.

## Discussion

The primary aim of this systematic review with meta-analysis was to examine the effects of PJT compared with active, passive or intervention controls on the maximal strength (i.e., dynamic, isometric, and isokinetic) in soccer players, irrespective of age, sex or competitive level. A discussion of the main results follows.

Compared to controls, PJT interventions improved maximal strength, including dynamic (ES = 0.43, Fig. 1), isometric (ES = 0.58, Fig. 2), and isokinetic (ES = 0.51, Fig. 3) maximal strength/torque measures. These results confirm the positive effects of PJT on maximal strength that were reported in previous studies [12, 124]. PJT-related maximal strength improvement was accompanied by (but not limited to) increased electromyographic activity (e.g., increased recruitment of motor units) [37], muscle activation strategies (e.g., improved intermuscular coordination) [23], single-fiber functioning (e.g., increased force) [39], muscle–tendon architecture (e.g., increased muscle pennation angle) [40], and increased muscle mass [125]. Maximal strength is related to sprint and jump performance [16, 126], common actions before scoring a goal in soccer [127] and associated with team positioning in the league [128]. Further, more competitive players exhibit greater maximal strength levels compared to less competitive players [129]. Therefore, improved maximal strength after PJT might contribute to soccer players' on-field performance. Performance enhancement through PJT interventions has proven effective and safe (with few reported injuries in the scientific PJT literature). In addition, PJT is an easy-to-administer training modality that can be conducted on the pitch and needs little equipment [24]. However, according to the GRADE assessment (Table 5), the certainty of evidence was considered low to very low for the main analyses. Moreover, the observed results were mainly assessed in male soccer players, as females represent a minor portion of the total soccer population. Therefore, a robust recommendation regarding the use of PJT to improve maximal strength in female soccer players is currently precluded. Although some limitations are difficult to address, future studies should provide stronger evidence by including larger samples in randomized-controlled trials.

The moderator analyses and the meta-regression analysis were available for maximal dynamic strength only. No moderator or meta-regression effect was noted for participants’ age (i.e., adults vs. youth). Participants were aged up to 25.0 years. Of note, a relatively greater number of studies reported the assessment of maximal strength indices for adult compared to youth players. For example, dynamic, isometric, and isokinetic maximal strength/torque was assessed in 9:9, 3:7, and 2:5 studies in youth:adult players, respectively. This contrasts with the considerably greater number of studies that reported athletic indices such as jumping and sprinting in youth compared to adult soccer players [29]. The reason why maximal strength was relatively unexplored in studies conducted in youth is unclear, but may include logistical reasons. For example, it may be difficult and time-consuming to perform safe and reliable maximal strength tests in youth players without experience in such procedures [130]. Indeed, when describing the training practices of academy soccer players, it has been noted that only 7–14% of their weekly training load is attributed to work in a gym [131]. Another reason might include fear of injury, or difficulty accessing sophisticated measurement equipment (e.g., isokinetic device) [132]. Irrespective of the potential reasons, future studies should include maximal strength assessment, given the potential of PJT to improve this outcome, and its relevance for soccer performance [14, 16]. Indeed, strength and PJT have been suggested to be important in youth soccer, due to associations with both physical performance [133–136] and injury prevention [33, 34, 137].

The moderator analyses and the meta-regression analyses for maximal dynamic strength also showed no moderator effect for total PJT programming duration (i.e., < 8 vs. ≥ 8 weeks) or total number of PJT sessions (i.e., < 16 vs. ≥ 16 sessions). The lack of differences in the PJT effects on maximal dynamic strength after longer compared to shorter interventions (e.g., < 8 vs. ≥ 8 weeks; < 16 vs. ≥ 16 sessions) are somewhat unexpected. As our moderator analysis involved cut-off values (categorical analysis), this may have affected the results. In a secondary analysis, we performed comparisons between study duration and total sessions using a continuous analysis approach. However, again, no significant differences were found. Considering the high heterogeneity for the maximal dynamic strength results noted after PJT (i.e., I^2^ = 77.9%), this may mean that interventions between studies varied not only in duration and total sessions, but in other potentially relevant PJT prescription variables, including exercise intensity (poorly reported among the included studies). However, currently there is no robust evidence to suggest a minimal effective duration or total number of sessions of PJT for the improvement of maximal dynamic strength in soccer players [29]. Alternatively, maximal strength response adaptation to PJT may be different from that of other physical fitness outcomes such as jump height and linear sprint speed [12, 88, 138]. Indeed, a correlation between PJT duration, sprint and jump performance improvement has been reported, although not for maximal strength [12, 88, 138].

Recently, there has been a proliferation of published studies on the effects of PJT in male soccer players [29]. However, there have been relatively few studies conducted with female soccer players [67, 121] and, according to the authors’ knowledge, none in master athletes. Indeed, of the included studies in the meta-analyses, only one study reported maximal strength data for females, in a mixed sample of male and female soccer players [121]. Furthermore, among the included studies in our meta-analyses, no youth female player data were available. This contrasts with the relatively greater number of studies conducted in youth males (i.e., 13 of a total of 27 meta-analysed studies). Therefore, the typical (not justified) sex imbalance in sport science publications [35, 139–146] is also noted in the context of this systematic review. The reason why females are less involved in PJT research and maximal strength measurements is probably multifactorial. A discussion of the (societal) biases that may underlie this phenomenon is beyond the scope of our review, and has been previously addressed [140]. Briefly, likely reasons could be (i) the lower incorporation of females in professional sports (e.g., soccer), (ii) cultural and/or religious reasons, and (iii) reduced research in females, retarding the transference to practice, as the potential PJT benefits could be less recognised by coaches (i.e., it might take up to 17 years until research findings are translated into practice) [147]. Soccer is a very popular sport around the world, with nearly 270 million people actively playing and a 50% increase in the number of female players observed between 2000 and 2006, with a stated aim for the sport to reach 60 million female players by 2026 [148]. With the increased participation of females in sports, research is required to enhance knowledge with regards to PJT programming for maximal strength optimization for female athletes.

Although six studies reported adverse health events (e.g., injury, illness) [65, 66, 99, 100, 111, 115] these were not directly linked to the PJT interventions. Thus, PJT seems a safe training method for soccer players aiming to improve maximal strength performance. Strength adaptations related to reduced injury risk were noted in some studies. In youth males (age, ~ 17 years) [122], the ratio of dominant leg/non-dominant leg peak torque for the knee extensors and flexors improved (~ 10%) after five weeks of training. Among adult males (age, ~ 21 years), eight weeks of training improved the hamstring eccentric:quadriceps concentric ratio, although only after unloaded jump training (7%) compared to loaded jumps (1%) [98]. Another study with adult males (age, ~ 23 years) [115], after seven weeks of training, noted significant improvements (5–10%) in hamstring/quadriceps peak concentric torque ratio in the dominant and non-dominant leg, and in the hamstring eccentric:quadriceps concentric peak torque ratio in the dominant and non-dominant leg. Reduced injuries are related to team success (e.g., position of the team in the league) [128]. However, among the studies (n = 24) that did not report soreness, pain, fatigue, injury, damage, or related adverse health effects, it is unclear if the lack of reporting was due to a true absence of adverse effects or merely due to the omission (i.e., non-deliberate) of such information in the reporting of the study methods. Moreover, some studies did not include in the methods section a description of instruments and protocols aimed to measure health effects (e.g., visual analogue pain scales; rating of perceived effort). Finally, these are surrogate outcomes and not direct measures of injury risk, and so they require careful interpretation. Future studies should elucidate the safety of PJT in soccer players by including robust measurement protocols and reporting methods.

### Limitations

A study limitation is the reduced number of studies available to perform moderator analyses regarding PJT programming variables, players’ sex, and players’ competitive level.

## Conclusions

Interventions involving PJT are more effective to improve maximal strength in soccer players compared to active, passive or intervention controls conditions involving traditional sport-specific training. This conclusion is derived from 27 studies with low-to-some risk of bias, low-to-high study heterogeneity, and low-to-very low certainty of evidence (GRADE rating), comprising 1,274 participants. The observed PJT-related maximal dynamic strength changes were similar for youth and adult players, and after shorter compared to longer interventions (i.e., < 8 vs. ≥ 8 weeks or < 16 vs. ≥ 16 sessions). Of the included studies, it seems that a minimal initial effective PJT dosage may involve one weekly training session for at least 5 weeks, involving ~ 70 jumps per session (usually at maximal intensity). However, the low-to-very low confidence in the available body of evidence precludes a robust recommendation for the implementation of PJT to improve maximal strength in soccer players.

### Supplementary Information

**Additional file 1**. Search strategy code line for each database.

## Data Availability

All data generated or analysed during this study are included in the article as Table(s), Figure(s), and/or Electronic Supplementary Material(s). Any other data requirement can be directed to the corresponding author upon reasonable request.

## Abbreviations

CI: Confidence interval
ES: Effect size
GRADE: Grading of Recommendations, Assessment, Development and Evaluation
I^2^: Impact of statistical heterogeneity
1-RM: One-repetition maximum
OSF: Open Science Framework
PICOS: Participants, intervention, comparators, outcomes, and study design
PEDro: Physiotherapy Evidence Database
PJT: Plyometric jump training
PRISMA: Preferred Reporting Items for Systematic reviews and Meta-Analyses
SCC: Stretch–shortening cycle
