# Supplementary material for: Plyometric Jump Training Effects on Maximal Strength in Soccer Players: A Systematic Review with Meta-analysis of Randomized-Controlled Studies
Source: Sports Med Open. 2024 May 10;10:52. doi: 10.1186/s40798-024-00720-w (PMC11087442; doi:10.1186/s40798-024-00720-w)
Supplement: Supplementary file 1 — Additional file 1. Search strategy code line for each database. [file 40798_2024_720_MOESM1_ESM.docx]

**Electronic Supplementary Material Table S1**

| Table S1  Search strategy code line for each database. | |
| --- | --- |
| **Databases** | PubMed, WOS (Core Collection) ^a^, Scopus. |
| **Keywords** | “ballistic”, “complex”, “cycle”, “explosive”, “force”, “jump”, “plyometric”, “power”, “shortening”, “stretch”, “training”, “velocity”. |
| **Database fields for the search** | PubMed and WOS: all ^b^; Scopus: title, abstract, keywords ^b^. |
| **Examples of search strategy code line** | PubMed: "plyometric exercise"[MeSH Terms] OR ("plyometric"[All Fields] AND "exercise"[All Fields]) OR "plyometric exercise"[All Fields] OR ("plyometric"[All Fields] AND "training"[All Fields]) OR "plyometric training"[All Fields]  WOS: ALL=plyometric and ALL=training  Scopus: TITLE-ABS-KEY ( plyometric AND training ) |
| ^a^: except for the keyword’s “jump”, “explosive”, and “power”, searched in all WOS databases.  ^b^: except for the keywords “jump”, “explosive”, and “power”, searched in the database field TITLE (a very poor efficiency was obtained in the search for results with the incorporation of other database fields). | |
